# Analytic validity of DecisionDx-Melanoma, a gene expression profile test for determining metastatic risk in melanoma patients

**DOI:** 10.1186/s13000-018-0690-3

**Published:** 2018-02-13

**Authors:** Robert W. Cook, Brooke Middlebrook, Jeff Wilkinson, Kyle R. Covington, Kristen Oelschlager, Federico A. Monzon, John F. Stone

**Affiliations:** 1 Castle Biosciences, Inc., 820 S. Friendswood Dr., Suite 201, Friendswood, TX 77546 USA; 2 3737 N. 7th St. #160, Phoenix, 85014 Phoenix USA

**Keywords:** Gene expression profiling, DecisionDx-Melanoma, Cutaneous melanoma, Metastasis, Analytic validity, Technical success

## Abstract

**Background:**

The DecisionDx-Melanoma test provides prognostic information for patients with cutaneous melanoma (CM). Using formalin-fixed paraffin-embedded primary tumor tissue, the RT-PCR-based test classifies patients into a low- (Class 1) or high-risk (Class 2) category for recurrence based on expression of 31 genes. The current study was designed to assess the analytical validity of this test.

**Methods:**

Inter-assay, inter-instrument, and inter-operator studies were performed to evaluate reliability of the 31-gene expression test results, sample stability and reagent stability. From March 2013 through June 2016, the gene expression test was performed on 8244 CM tumors. De-identified data from Pathology Reports were used to assess technical success.

**Results:**

Robust sample and reagent stability was observed. Inter-assay concordance on 168 specimens run on 2 consecutive days was 99% and matched probability scores were significantly correlated (R^2^ = 0.96). Inter-instrument concordance was 95%, and probability scores had a correlation R^2^ of 0.99 (*p* < 0.001). From 8244 CM specimens submitted since 2013, 85% (7023) fulfilled pre-specified tumor content parameters. In these samples with sufficient tumor requirements, the technical success of the test was 98%.

**Conclusion:**

DecisionDx-Melanoma is a robust gene expression profile test that demonstrates strong reproducibility between experiments and has high technical reliability on clinical samples.

**Electronic supplementary material:**

The online version of this article (10.1186/s13000-018-0690-3) contains supplementary material, which is available to authorized users.

## Background

Clinical staging of cutaneous melanoma (CM) is based upon clinicopathologic parameters such as tumor thickness, ulceration, mitotic rate, and sentinel lymph node (SLN) status [1]. SLN status, as determined by SLN biopsy (SLNB), has the greatest prognostic significance of established factors [2–4]. The majority of CM patients are initially diagnosed with early stage (I or II) disease and have favorable prognosis [1, 5]. However, a substantial percentage of early stage patients develop metastases and two-thirds of all melanoma-related deaths occur in patients initially diagnosed with Stage I or II disease [1, 5–7]. Accurate methods for predicting metastatic risk are, therefore, of paramount importance for implementing risk appropriate management plans to enable early identification of disease progression and timely intervention with current treatment options.

We have developed and validated a gene expression profile (GEP) test that assesses melanoma tumor biology to improve the prediction of metastasis risk beyond traditional clinicopathologic factors [8, 9]. The GEP test employs RT-PCR gene expression analysis to evaluate the expression of 31 gene targets using primary tumor biopsy tissue and provides a binary classification of low risk (Class 1) or high risk (Class 2) of metastasis within 5 years. Clinical validation studies have shown the test to be an accurate prognosticator that is independent of AJCC staging criteria [8, 9]. Molecular classification was shown to improve risk prediction when used in combination with SLNB, identifying as Class 2 more than 80% of SLN-negative patients who developed metastatic disease and died from melanoma [8].

In this study, we report the analytic validity of the GEP test, including reproducibility (inter-assay, inter-instrument, and inter-operator concordance) of molecular classification and technical reliability of clinical testing in accordance with published guidelines [10, 11], when performed in a College of American Pathologists (CAP)-accredited, Clinical Laboratory Improvement Act (CLIA)-certified laboratory setting.

## Methods

### Sample and clinical data collection

All samples were acquired through routine clinical testing of primary CM tumors with the 31-GEP test. Ten 5-μm tissue sections were cut from the formalin-fixed, paraffin-embedded (FFPE) block containing the primary melanoma tissue (biopsy or wide local excision). The first recut slide was stained with hematoxylin and eosin (H&E) and the 9 subsequent slides remained unstained. The slides were sent to Castle Biosciences’ centralized CAP-accredited, CLIA-certified laboratory. All analyses were performed using de-identified technical and pathology report data. Therefore, Institutional Review Board approval was not required because this analysis is exempt from the regulatory review requirements as set forth in section 46.101 (b) of 45 CFR 46.

### RT-PCR analysis and risk assignment

The H&E-stained slides for all research and clinical samples are reviewed by a licensed pathologist for (a) confirmation of the presence of primary melanoma tumor and (b) marking an area containing sufficient tumor density (initially ≥60% and subsequently lowered to ≥40%). On the unstained slides, this tumor tissue was macrodissected and cDNA was converted from total RNA as previously described [9]. Quantitative real-time PCR was performed on the 7900HT Fast Real Time PCR System (Life Technologies) or the QuantStudio OpenArray system (Life Technologies). Cards or OpenArrays contained primers specific for 28 class-discriminating gene targets and three endogenous control genes [9].

**Fig. 1:**
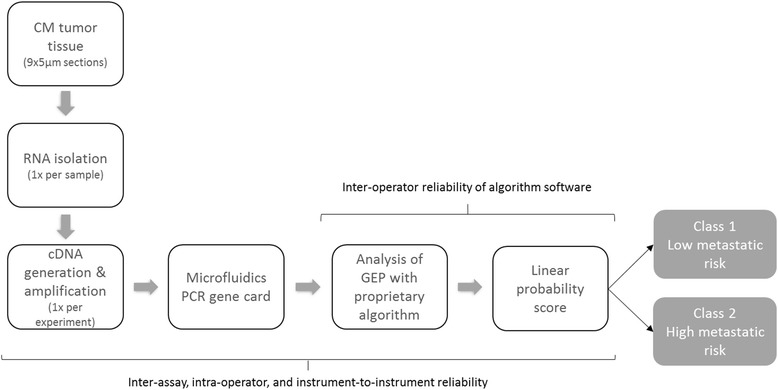
Workflow schematic of the DecisionDx-Melanoma test. Steps in the performance of the test are presented along with the corresponding reliability analyses

Research cases used for stability studies had RNA extracted only once per sample (Fig. 1). RNA stability studies, therefore, compared assays run from the same isolated RNA sample that were used immediately or stored at -80^o^. New FFPE fixed slides were not obtained for any cases, and subsequent reliability and reproducibility experiments were performed beginning with the cDNA generation and amplification step. Fresh samples were not processed to perform the algorithm software reliability studies.

Resulting standardized ΔCt values for each test sample were analyzed with a radial basis machine (RBM) predictive model from a validated training set of 164 melanoma cases with known metastatic outcomes [9] using JMP Genomics SAS-based software (SAS, Cary, NC). RBM modeling provides a qualitative binary classification of Class 1 (low risk) or Class 2 (high risk) tumor biology based on a quantitative linear probability score from 0.0 to 1.0, with a score of 0.5 being the cutoff between the binary classes. A normal confidence interval for each Class is established by using one SD from the median score of the training set cases without recurrence (0.0–0.41) or with recurrence (0.59–1.0); scores falling outside this range are considered of reduced statistical confidence (0.41–0.5; 0.5–0.59). Results are reported as Class 1A (0.0–0.41), Class 1B (0.41–0.5), Class 2A (0.5–0.59) and Class 2B (0.59–1.0).

### Statistical analysis

Statistical analysis was performed using Microsoft Excel and WinSTAT for Microsoft Excel version 2012.1 (R. Fitch Software, Cambridge, MA) and/or the R package. Analytic validity and reliability was reported as 1) the qualitative concordance of RBM binary class assignment (Class 1 or Class 2), 2) the qualitative concordance of RBM subclass assignment (Classes 1A, 1B, 2A or 2B) and 3) correlation of quantitative probability score values. Association of clinical factors with test outcomes was primarily determined using Fisher’s exact and χ-squared tests, with other tests indicated where appropriate.

## Results

### Specimen and sample stability

To assess RNA stability, results for 21 samples were obtained on 2 separate days, 3 months apart. Complimentary DNA was generated for each of the two experiments using the original isolated RNA sample. Comparison of probability score values (range 0.0–1.0) showed high correlation (R^2^ = 0.99, *p* < 0.001), 100% concordance in binary risk classification (Class 1 or Class 2) and 90% concordance on subclass risk classification (Class 1A, 1B, 2A, or 2B) (18 of 21 cases; 95% confidence interval [CI] = 70–99%). Analysis was also performed on an additional 20 samples tested on 2 separate days at intervals ranging from 48 to 122 days apart. Again, probability score values were highly correlated (R^2^ = 0.96, *p* < 0.02) with 100% concordance (95% CI = 83–100%) in binary class assignment. Risk classification using normal and reduced confidence subclasses was concordant in 18 of 20 (90%) cases (95% CI = 68–99%).

**Fig. 2:**
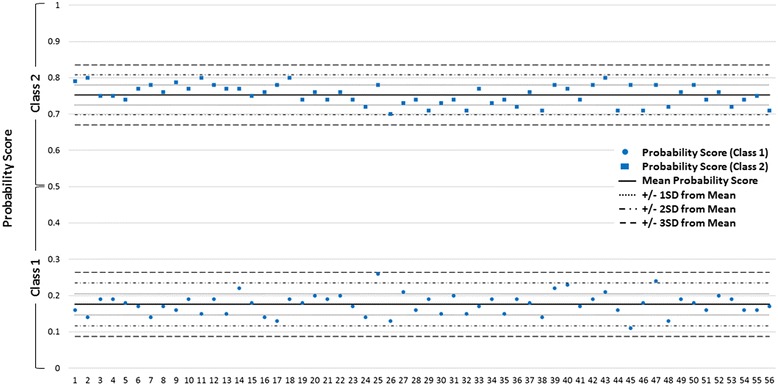
Levey-Jennings analysis for positive control samples across 56 experiments. Reproducibility of assay performance for one Class 1 and one Class 2 positive control cDNA sample is shown. Probability scores were recorded from experiment to experiment over a 3 month period and across multiple lots of reagents

To evaluate long-term cDNA stability, we monitored reproducibility of assay performance for one Class 1 and one Class 2 cDNA sample (positive controls) included from experiment to experiment and across multiple lots of reagents (Fig. 2). Two negative water controls without template were also included with each OpenArray run over a 3-month period in which 56 assays were performed. No assays were rejected due to amplification in the negative controls. The Class 1 positive control sample had a mean quantitative probability score of 0.176 (SD = 0.029, 2SD = 0.059 and 3SD = 0.088) and the Class 2 positive control sample had a mean probability score of 0.752 (SD = 0.027, 2SD = 0.055, and 3SD = 0.082), reflecting robust assay repeatability.

Short-term cDNA stability was also evaluated. Ten samples underwent reverse transcription and the cDNA was stored for 96 h per standard operating procedures; RNA from the same 10 samples was then reverse transcribed on the day the assay was performed. All samples were run on a single assay and the resulting probability scores from the two groups were compared. We found probability score values to be significantly correlated (R^2^ = 0.89, *p* < 0.05), and both subclass and binary risk classifications were 100% concordant (95% CI = 69–100% for both).

**Fig. 3:**
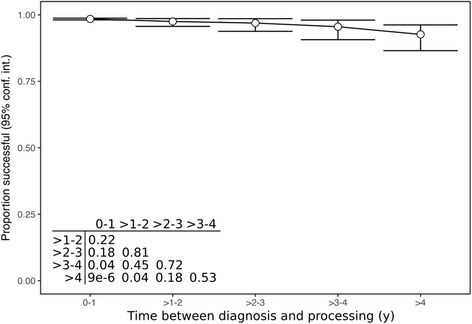
Success rates for the DecisionDx-Melanoma test for FFPE samples at various time points after diagnosis. Processing times are in years since diagnosis. Error bars represent 95% confidence intervals for the proportion of successful GEP tests in the indicated bin. Inset table represents the adjusted (FDR) *p*-values of Chi-squared comparisons between the indicated time bins

To further examine sample stability, the success rates of GEP processing at various time points after diagnosis were assessed. We examined a total of 6772 FFPE-derived samples with documented age of specimen that were stored for up to 1 year, 1–2 years, 2–3 years, 3–4 years, or greater than 4 years prior to GEP testing. Overall we observed 98% (6647 of 6772) success rate in all specimens. There was a slight decrease in success rates in samples that had been stored for longer periods of time (*p* < 0.0001; Fig. 3).

We also examined the effect of delay in sample processing on the stability of the 31-gene GEP assay. We evaluated outcomes in 275 retrospective research samples processed 1.5 to 16 years after diagnosis in which a significant association between GEP Class and recurrence-free survival has been previously published [8, 9]. Multivariate Cox regression model to evaluate the interaction between GEP Class and time to sample processing showed no effect of delay in processing time (*p* = 0.25) and no statistical interaction between the sample age and GEP Class covariates (*p* = 0.51) was observed. These data indicate that the delay in processing time does not alter association of Class assignment and recurrence risk.

### DecisionDx-Melanoma assay reliability

**Fig. 4:**
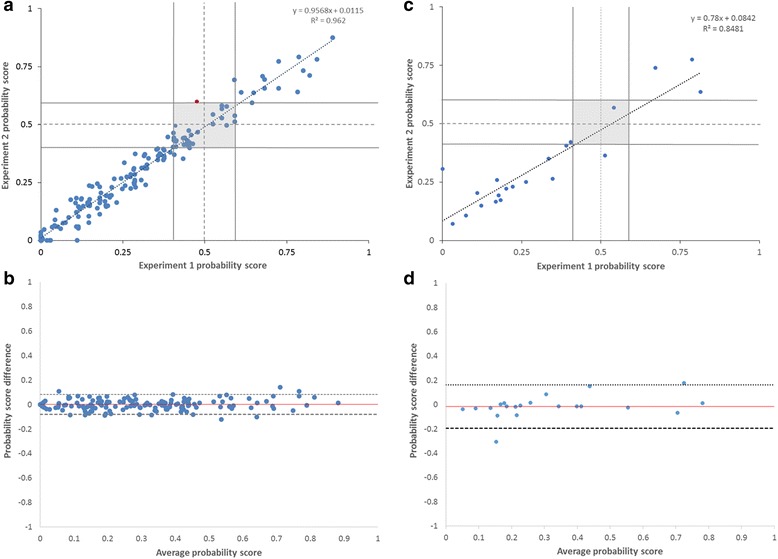
Results from concordance studies on probability score values of the DecisionDx-Melanoma test. **a** Inter-assay correlation analysis for 168 cases; **b** Bland-Altman plot for 168 cases showing estimated bias (mean difference in probability scores, red line) and 95% confidence interval (dashed lines); **c** instrument-to-instrument correlation analysis for 21 cases; **d** Bland-Altman plot for 21 cases showing estimated bias (mean difference in probability scores, red line) and 95% confidence interval (dashed lines)

To assess inter-assay reliability of the 31-gene expression profile test, results were obtained on two separate days for 168 clinical melanoma samples. The time interval between the testing of matched samples ranged from 1 day to greater than 6 weeks. A total of 44 clinical samples were analyzed using the 7900HT Real-Time PCR System, and 124 samples were analyzed using the QuantStudio Real-Time PCR System. Comparison of probability score values (range 0.0–1.0) resulted in highly correlated scores (R^2^ = 0.96, *p* < 0.001; Fig. 4a). Binary risk classification was concordant for 167 of 168 (99%, 95% CI 96–100%) cases and subclassification was concordant for 155 of 168 (92%, 95% CI 87–96%) cases. The single case changing from Class 1 to 2 generated probability scores close to the 0.5 cutoff in the first run (0.476). Overall, the mean absolute difference in matched probability scores was 0.03 and showed 95% of variability to be within acceptable limits and not likely to change class assignment, as determined by Bland–Altman analysis (Fig. 4b).

We evaluated intra-assay reliability by obtaining results from 7 samples run in triplicate on a single OpenArray plate. The process was repeated on 3 separate runs for a total of 21 samples. Binary classification resulted in 100% concordance (95% CI 94–100%) while subclassification resulted in 98% concordance (62 of 63; 95% CI 91–100%).

Lot to lot variability for critical reagents has been evaluated in experiments ranging from 4 to 19 samples and with 2–6 reagent lots. Correlation of discriminant scores was above 0.96 for all experiments, with binary class concordance of 100% in all cases and subclass concordance above 90% for all but one reagent, in which a subclass concordance of 75% was achieved based on only one of four samples being discrepant (Additional file 1: Table S1).

Inter-platform reliability was assessed by comparing probability scores generated from 21 samples tested on both the 7900HT and QuantStudio systems. The results indicated significant correlation of probability score values between the two systems (R^2^ = 0.85, *p* < 0.001; Fig. 4c), and concordant subclass prediction was observed for 95% of cases (19 of 21). One of the matched probability score values generated for each of the two discordant cases was in the reduced confidence range (0.421, Class 1B and 0.513, Class 2A). The mean absolute difference in probability scores between instruments was 0.06 (Fig. 4d).

Twenty-two samples were run on two different QuantStudio instruments and the resulting probability scores were compared to evaluate inter-instrument reliability. Probability score values were highly correlated (R^2^ = 0.99, *p* < 0.001) and binary classification was concordant in 21 of 22 (95%) cases (95% CI 88–100%). The mean absolute difference in probability score values between instruments was 0.02.

### Inter-operator reproducibility of the predictive modeling algorithm

To evaluate inter-operator reliability of the JMP Genomics predictive modeling software, RBM analysis of gene expression data for 268 clinically tested melanoma samples was performed separately by two personnel on multiple days. Quantitative probability scores generated by both analyses were identical (R^2^ = 1.0, *p* < 0.001; data not shown), and qualitative subclass and binary class prediction was concordant for all 268 cases (100%).

### DecisionDx-Melanoma technical experience

**Fig. 5:**
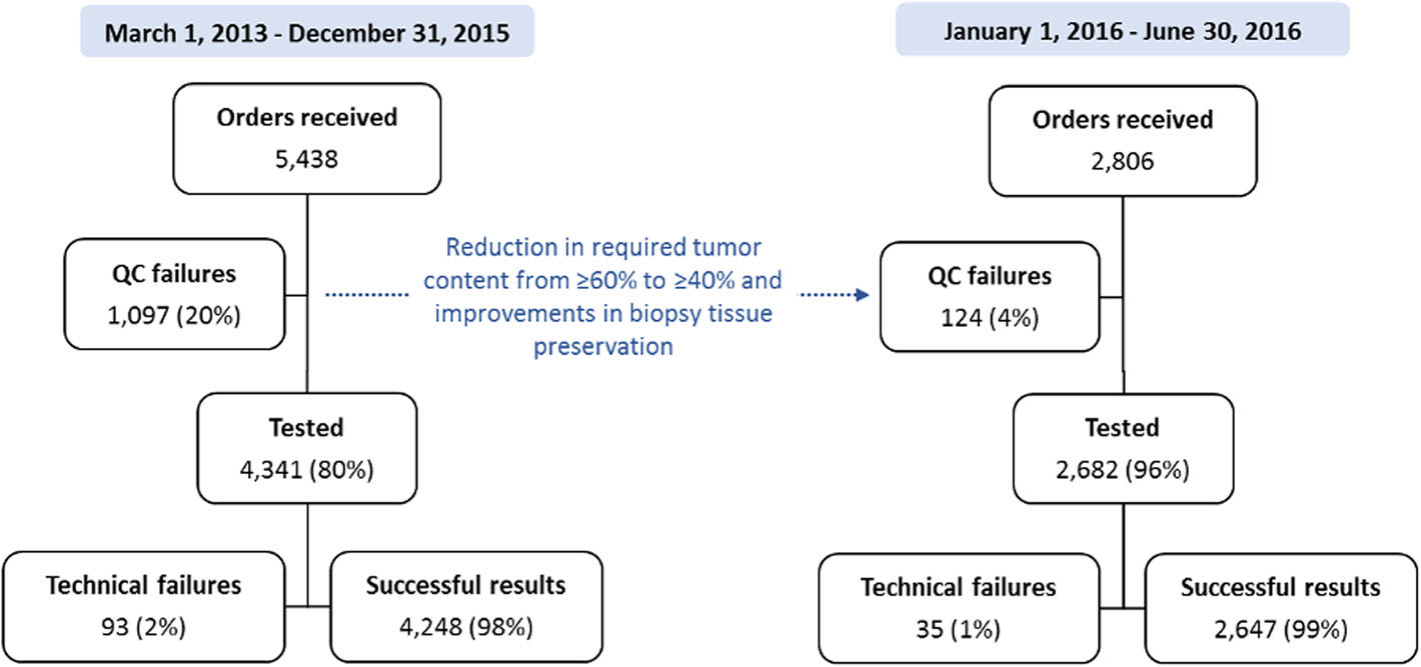
Technical experience of the DecisionDx-Melanoma test for samples submitted from March 2013 to June 2016. Quality control rejections, technical failures and successfully tested samples are presented for the time periods before and after the reduction in required tumor density, implemented after successful clinical validation and through educational efforts to improve biopsy tissue preservation at the dermatopathology level

From March 1, 2013 through June 30, 2016, DecisionDx-Melanoma testing was requested for 8244 primary melanoma cases from 1123 centers in the United States and Spain. Samples submitted for DecisionDx-Melanoma testing must have a sufficient density of tumor cells in order to proceed with gene expression profiling. Of the 8244 specimens, 1221 (15%) had insufficient tumor content for testing. As shown in Fig. 5, 90% of the 1221 samples with insufficient tumor density were submitted during the period from March 1, 2013 to December 31, 2015, reflecting a 20% rate of insufficient tissue for testing. Quality control studies completed in March 2015 permitted a decrease in the required tumor content from ≥60% to ≥40% melanoma within a macro-dissectible area of the tissue section. This, coupled with efforts to improve biopsy tissue preservation at the local processing level (including educational outreach to pathology laboratory staff, pathologists, and ordering clinicians), resulted in a dramatic reduction in the number of insufficient specimens. From January 1, 2016 to June 30, 2016, only 4.4% (124 of 2806) of samples lacked sufficient tumor content, reflecting 78% reduction in quality control rejections compared to the previous period (Fig. 5). No changes in the proportion of thin tumor (≤1 mm Breslow thickness) cases was observed in this period.

Overall, 98% (6895 of 7023) of cases submitted with sufficient tumor volume were successfully tested and reported, with only 1.8% cases having a reported technical failure due to amplification failure in control and/or prognostic genes. The technical success rate increased to 99% (2647 of 2682) for the period of January 1, 2016 to June 30, 2016 (Fig. 5).

## Discussion

As precision medicine in oncology strongly relies on accurate molecular classification of tumors, it becomes imperative to determine the reliability and accuracy of molecular tests. Groups such as the Evaluations of Genomic Applications in Practice and Prevention (EGAPP) Working Group and National Comprehensive Cancer Network (NCCN) have recognized three integral components of molecular diagnostic and prognostic tests: analytic validity, clinical validity, and clinical utility [11, 12]. The GEP test described in this study has been clinically validated in three multicenter studies, showing that molecular class assignment is able to accurately and reliably identify CM patients who have a high risk of developing metastases [8, 9, 13], and its clinical utility was recently reported in a study showing that physicians directed their management choices based on patients’ GEP risk classification [14]. Here we aimed to report the analytic validity of the GEP using recognized measures of reliability.

Inter-assay, inter-operator, and inter-instrument reliability, measured using both the quantitative probability scores and binary classifications of risk, met or exceeded the requirements for a clinically applied prognostic test [12, 15, 16]. Probability scores were highly correlated when the same samples were tested on different days or experiments were performed on different machines (R^2^ = 0.96 and 0.85, respectively), and concordance of binary class prediction was strong for both analytic parameters (99% and 95%, respectively). These results highlight the strength of the protocols used to perform the 31-gene test, and the reproducibility of results when the test is run in a CAP-accredited, CLIA-certified central laboratory.

The majority of CM tumors are diagnosed when they are < 1 mm in thickness [6, 7]. As such, the amount of tumor tissue for diagnostic and prognostic testing can be limited and preservation of that tissue is an important consideration in the management of CM patients. Tumor tissue preservation is increasingly a priority as biomarker testing, such as BRAF mutational analysis, PD-L1 immunohistochemistry and this GEP test, are integrated in patient management decisions [17–19]. The 98% technical success rate of the test indicates consistent high performance using available tumor biopsy tissue and compares favorably to the performance of other genomic classifier tests performed on FFPE specimens [20]. This result is achieved despite the fact that clinical specimens were submitted by over 1100 institutions submitting clinical samples, and reflects robustness regardless of institution-specific tissue processing protocols and shipment variables. Our results also highlight the importance of communication between laboratories, as the number of specimens with insufficient tumor tissue was dramatically improved by implementing direct communication with dermatopathologists to improve biopsy tissue preservation measures. As shown in Fig. 5, the result is that 96% of submitted specimens were clinically tested from January 1 through June 30, 2016. As there is increased recognition of the value of molecular testing, it is important to develop sample preservation protocols and robust molecular tests that will enable the clinical application when limited tissue is available.

While there is expected correlation between the GEP prediction of risk and AJCC staging, there is no perfect correlation. We know that, despite having a low population-based risk, the majority of patients that die from melanoma are initially diagnosed with stage I or II disease [1, 5–7]. Previous studies have shown that the GEP test a) is independent from the standard clinicopathologic staging parameters, b) adds additional information about recurrence/metastasis risk and, c) is able to identify up to 90% of Stage I and II patients who die from their disease as high-risk (Class 2) [8, 9].

## Conclusions

These results demonstrate that the 31-gene expression profile test is a precise, reliable and technically robust molecular test. Using a framework of accepted criteria to establish analytic validity, we present strong test performance and reproducibility. Taken together, the results of this analysis and previous clinical validation studies show that the GEP prognostic test is a robust and clinically useful tool to implement risk-appropriate healthcare decision-making in CM patients.

## Additional file

Additional file 1: **Table S1** Lot-to-lot stability of reagents used to run the DecisionDx-Melanoma test. (DOCX 14 kb)

## Abbreviations

AJCC: American Joint Committee on Cancer
CAP: College of American Pathologists
CI: Confidence interval
CLIA: Clinical Laboratory Improvement Act
CM: Cutaneous melanoma
EGAPP: Evaluations of Genomic Applications in Practice and Prevention
FFPE: Formalin-fixed, paraffin-embedded
GEP: Gene expression profile
H&E: Hematoxylin and eosin
NCCN: National Comprehensive Cancer Network
RBM: Radial basis machine
SD: Standard deviation
SLN: Sentinel lymph node
SLNB: Sentinel lymph node biopsy
