# Supplementary material for: Analytic validity of DecisionDx-Melanoma, a gene expression profile test for determining metastatic risk in melanoma patients
Source: Diagn Pathol. 2018 Feb 13;13:13. doi: 10.1186/s13000-018-0690-3 (PMC5809902; doi:10.1186/s13000-018-0690-3)
Supplement: Supplementary file 1 — Table S1 Lot-to-lot stability of reagents used to run the DecisionDx-Melanoma test. (DOCX 14 kb) [file 13000_2018_690_MOESM1_ESM.docx]

Additional file 1: Table S1. Lot-to-lot stability of reagents used to run the DecisionDx-Melanoma test

| Reagent | # of samples | # of runs | # of days | # of lots | R^2^ | Subclass Concordance | Binary Class Concordance |
| --- | --- | --- | --- | --- | --- | --- | --- |
| Taqman CM OpenArray | 23 | 2 | 2 | 3 | 0.97 | 100% | 100% |
| Deparaffination buffer | 14 | 2 | 2 | 4 | 0.98 | 93% | 100% |
| DNAse I | 14 | 2 | 2 | 2 | 0.98 | 93% | 100% |
| Gene Expression Master Mix | 7 | 2 | 2 | 2 | 0.96 | 100% | 100% |
| OpenArray Master Mix | 14 | 3 | 3 | 6 | 0.97 | 93% | 100% |
| Pre-Amp Master Mix | 19 | 4 | 4 | 4 | 0.97 | 95% | 100% |
| Proteinase K | 4 | 1 | 1 | 2 | 0.98 | 75% | 100% |
| Proteinase K buffer | 4 | 1 | 1 | 2 | 0.99 | 100% | 100% |
| Qia kit | 14 | 2 | 2 | 2 | 0.99 | 93% | 100% |
| RNAse inhibitor | 3 | 1 | 1 | 2 | 1.00 | 100% | 100% |
| RT kit | 8 | 2 | 2 | 3 | 0.97 | 100% | 100% |
